# How Long Halt Ye?

**Published:** 1920-11-27

**Authors:** 


### The Hospital

The Workers' Newspaper of Administrative Medicine and Institutional Life, Administration, National Insurance and Health.

No. 1799, Vol. LXIX. SATURDAY, NOVEMBER 27, 1920. PRICE TWOPENCE

### HOW LONG HALT YE?

Now that patients' payments have become established, and weekly contributions seem likely to extend generally in London, less sympathy than ever can be felt for those who declare their own bankruptcy, and appeal for unlimited State aid at every difficulty. It is admitted that patients' payments do not solve the whole problem, though at the London Hospital, where they are expected to yield about £20,000 a year, they have already secured, apparently in permanence, almost as much as was obtained by Lord Knutsford's last appeal. Still more must be done, if only to tide over the interval before reasonable hopes of extended weekly contributions can be realised. At this juncture arrives Mr. R. J. Coles' interesting suggestion that insured persons should be covered, by extra payments, for institutional treatment, the absence of which was the weakest point in the very weak Insurance Act. Like all such schemes, however, its accomplishment would involve further delay, in consequence of further legislation; and the question has to be decided whether such a scheme and extended weekly contributions can subsist side by side. It is possible that a choice may have to be made between them. Since, in the long run, an ounce of voluntary help is worth two ounces of State aid, we hope that extended contributions will be given a fair trial before Mr. Coles' suggestion is seriously entertained by the Government.

An immediate point, however, is to urge the need of a definite hospital policy. Of the proposals immediately before the hospitals, either extended weekly contributions should be encouraged, or the extension of insured persons' payments. It would be a tactical mistake to pursue both policies simultaneously, unless there were weighty evidence that they would not conflict with each other. We should like to hear Mr. R. J. Coles' views on this point. Does he think that patients' payments, weekly contributions, and a further charge on insured persons for institutional treatment can subsist together? If so, what are his arguments? If not, which would he prefer to sacrifice? Both (or is it all three?) schemes, however, especially the voluntary one, have the virtue of simplicity, and should be tried long before we come to the possibility of co-ordinating the voluntary hospitals and the Poor-law infirmaries under the County Council. Once again London is the weak spot, but, fortunately, Lord Knutsford seems alone in a disposition to throw up the sponge of London's largest hospital and abandon the cause to which his life's work has been devoted. He rather likes to startle the world with untempered announcements, and the virtue which this power of attracting attention achieves in one direction must be set against the disservice which it occasionally causes in another.

In the present day large sums are raised by multitudinous pennies, and we wish Lord Knutsford would show some sign of recognising this in other spheres beyond that of payments from patients, which, after all, was the easiest line for him. If he would put his shoulder to the wheel further in this direction, he would be doing a very great service indeed. As it is, if he will pardon us for saying so, he seems to have no ideas between personal appeals on the old lines and a complete abandonment of the old lines altogether. This attitude is neither statesmanship nor good sense. It is really a form of intellectual idleness. A policy is not born of a violent oscillation between two opposite extremes. Extremes meet at the centre of indifference, and the wise man makes the most of that centre. We therefore hope that Lord Knutsford will either devote himself to the encouragement of the movement for extended weekly contributions, or, if that is asking too much from his apparent scepticism of their value, that he will ally himself to Mr. Coles' scheme. Either, as we have already urged, converges on the point where State aid and the voluntary system meet; and that meeting place is the spot from which to evolve a policy, if the voluntary system is not lazily to be thrown away.

